# The Horton Infirmary, Banbury

**Published:** 1916-09-09

**Authors:** 


### SMALLER GENERAL HOSPITALS.

### The Horton Infirmary, Banbury.

An excellent instance of the small general hospital which is carrying on, in spite of the war and the competition in charitable giving, is the Horton Infirmary, Banbury, of forty beds, which since 1872 has done valuable work for the town and neighbourhood. Banbury is an industrial and market town of over 13,000 inhabitants, but the district served, although mainly agricultural in character, has a considerable ironstone quarrying industry, and this, with the railways, foundries, and factories in the town provide the infirmary with emergency cases, which in its absence would have to be sent to one of the county towns. It fills a large gap between the hospitals of Oxford, Leamington, and Northampton, each over twenty miles distant, and may be said to provide for an area comprising the town of Banbury and 180 villages.

Since the war the infirmary has been without a house surgeon, but the work has been most admirably carried on by the able matron, who possesses, besides professional qualities of a high order, the art of inspiring her staff with zeal and the public with confidence. The staffs of civil hospitals are rather apt to be overlooked in a time like the present, and public recognition that they are serving their country equally with those who have undertaken war nursing encourages them a great deal, although they seek it not. The honorary medical staff of the Banbury Infirmary have borne all the medical and surgical work during the war, and although overburdened with their own practices and the extra work which the absence of some of their *confrères* at the war has thrown upon them, have been unremitting in their attention to the needs of the infirmary. The infirmary is equipped with an up-to-date operating-theatre, an anæsthetic-room (the gift of a local resident), and an *x*-ray installation (given at a cost of £250 by the Banbury Co-operative Society). A great deal of surgical work is done there, and operations are performed which a few years ago necessitated cases being sent away to larger hospitals. There are private wards, and the infirmary now receives patients who previously had to go to a nursing home in one of the larger towns. The Horton Infirmary has also played a part in war work, for during the billeting of some battalions in the town it received many of their sick cases, and before the opening of a V.A.D. hospital in Banbury it emptied one of its wards and took overflow cases from the Southern Command Base Hospital in Oxford. It still does surgical work for the troops in the town.

### Pound Day Results.

An example of the encouragement which staffs of such hospitals as this might well be given at the present time has just occurred at this infirmary. Everyone knows with what heavy incidence housekeeping expenses now fall upon the domestic exchequer. They fall equally heavily on such institutions as the Horton Infirmary, which is entirely supported by voluntary contributions. With a view to lightening this charge Lady Evelyn Parker, an excellent neighbour and friend of the infirmary, inaugurated a "Pound Day," and appealed to the inhabitants of the town and district with a list of acceptable articles for the store-room, asking all benevolently disposed to send on August 25 a pound of one or the other of them. Such a magnificent response was made that, without counting vegetables, which came in by the basket-load, roughly speaking 4,000 lb. of goods poured in, and the store-room is fortified against war prices in many leading lines which, let us hope, will enable it to see the struggle through, while the treasurer is, of course, correspondingly relieved and is freer to listen to the requirements of the medical staff. In addition to the goods, Lady Evelyn Parker received no less than £88 in money, and the matron's visions of long-needed acquisitions, which the committee felt bound to defer, are showing a promise of rapid materialisation. Altogether the "Pound Day" has been a most cheering event to the staff and friends of the infirmary. The war claims on the generosity of the inhabitants have been considerable and have been generously met, but the experience of "The Horton," as it is locally known, shows that the public are still prepared to stand by their own hospitals, and often all that is required is some friend who will express its needs and propound some simple and practical way in which all can help to meet them.

As evidence of the general confidence felt in the Horton Infirmary, and of the fact that it is not left to be run entirely by the generous well-to-do, it may be mentioned that the Banbury and District Workpeople's Hospital Fund Association, which was started some six years ago, sends members to represent it on the committee of management, and organises a systematic collection from the workpeople of the town and district. Last year it paid £250 into the infirmary's funds, and since its formation the association has contributed £1,550.

The demands upon the infirmary have long outgrown the accommodation, and before the war a building fund was formed for its extension. When times become normal again it is hoped that the scheme, which includes a new out-patients' department, will be carried out.

